# Differential prioritization of intramaze cue and boundary information during spatial navigation across the human lifespan

**DOI:** 10.1038/s41598-021-94530-9

**Published:** 2021-07-27

**Authors:** Franka Glöckner, Nicolas W. Schuck, Shu-Chen Li

**Affiliations:** 1 Faculty of Psychology, Chair of Lifespan Developmental Neuroscience, Technische Universität Dresden, Zellescher Weg 17, 01069 Dresden, Germany; 2 Max Planck Research Group NeuroCode, Max Planck Institute for Human Development, 14195 Berlin, Germany; 3 Max Planck UCL Centre for Computational Psychiatry and Ageing Research, Berlin, Germany; 4 CeTI - Centre for Tactile Internet with Human-in-the-Loop, Technische Universität Dresden, 01069 Dresden, Germany

**Keywords:** Human behaviour, Spatial memory, Working memory, Hippocampus, Cognitive ageing, Cognitive neuroscience, Development of the nervous system, Neural ageing

## Abstract

Spatial learning can be based on intramaze cues and environmental boundaries. These processes are predominantly subserved by striatal- and hippocampal-dependent circuitries, respectively. Maturation and aging processes in these brain regions may affect lifespan differences in their contributions to spatial learning. We independently manipulated an intramaze cue or the environment’s boundary in a navigation task in 27 younger children (6–8 years), 30 older children (10–13 years), 29 adolescents (15–17 years), 29 younger adults (20–35 years) and 26 older adults (65–80 years) to investigate lifespan age differences in the relative prioritization of either information. Whereas learning based on an intramaze cue showed earlier maturation during the progression from younger to later childhood and remained relatively stable across adulthood, maturation of boundary-based learning was more protracted towards peri-adolescence and showed strong aging-related decline. Furthermore, individual differences in prioritizing intramaze cue- over computationally more demanding boundary-based learning was positively associated with cognitive processing fluctuations and this association was partially mediated by spatial working memory capacity during adult, but not during child development. This evidence reveals different age gradients of two modes of spatial learning across the lifespan, which seem further influenced by individual differences in cognitive processing fluctuations and working memory, particularly during aging.

Navigating through spatial environments is a ubiquitous daily activity for animals and humans of different ages. In addition to internal sensory information arising from self-motion, spatial navigation requires efficient integration, storage, and evaluation of different types of information that are available in the environment over time. These different types of information implicate multiple forms of spatial representations and, hence, parallel involvement of different brain systems. The hippocampal formation plays an important role in forming complex mental representations of spatial layouts^1,2^ and providing information about direction based on boundary-sensitive signals^3^. In comparison, learning to associate intramaze cues with specific behavioral responses (e.g., turning at a cued intersection) relies more on the striatal network^4^. In line with findings from animal research, functional magnetic imaging studies suggest that whereas learning object locations relative to the environmental boundary correlates more with hippocampal activity, inferring object locations from an intramaze cue correlates with activity in the striatum^5–7^. Moreover, the medial prefrontal cortex (mPFC) may play a role in the top-down regulation of these two parallel forms of spatial learning. For example, the mPFC seems to be recruited when the hippocampus and the striatum are similarly activated or deactivated during spatial navigation^5^. Hippocampal-prefrontal interactions seem also relevant when future goals and locations need to be prospectively coded by hippocampal place cells during navigation^8^.

While the complex map-like representation of locations relative to spatial boundaries and simple associations between intramaze cues and responses may operate in parallel, their different computational demands based on the different complexity of their underlying learning rules^6^ are likely to interact differently with age-related and individual differences in neurocognitive resources. In consequence, their relative contributions to spatial learning might vary across the lifespan. Individuals who differ in the efficacies of these hippocampal and striatal functions may differ in their prioritization of using hippocampal-dependent boundary and striatal-dependent intramaze cue information during spatial navigation. Potential mechanisms affecting the efficacies of these hippocampal- and striatal-dependent functions involve, among other factors, brain maturation and aging processes as well as changes in the dopamine modulation of the prefrontal-hippocampal-striatal circuitry across the lifespan. Dopamine neurons in the ventral tegmental area project to the hippocampus, the ventral striatum and the prefrontal cortex, whereas dopamine projections from the substantia nigra reach to the dorsal striatum^9^. Early neuropharmacological studies investigating dopamine modulation of spatial navigation in rodents indicate that hippocampal and striatal^10–12^ but also prefrontal dopamine modulation^13,14^ is necessary for processing spatial information during navigation. Moreover, the dopamine system seems to be implicated in hippocampal-dependent plasticity and memory functions^15,16^. Computational and empirical evidence indicates an inverted U-shaped function relating dopamine signaling and cognitive functioning across the lifespan, with suboptimal dopamine levels (for example, during childhood and in older age) being associated with suboptimal cognitive performance^17–19^. At the neuronal level, animal studies have shown that the stability of hippocampal place fields and the specificity of spatial tuning of place cells develop only gradually and approach adult performance at an age period, which approximately corresponds to late childhood and early puberty in humans^20^. Furthermore, during development, place cell stability and accuracy appear only sufficient for navigation in close proximity to spatial boundaries as place cell firing seems mostly stabilized by signals from boundary-responsive cells given the protracted maturation of necessary grid cell networks^21^. In older age, the specificity of place cell firing declines with significant loss of functional synapses and altered calcium regulation in various hippocampal regions, which negatively affects hippocampal plasticity and memory functions^22,23^.

Consistent with the animal literature, studies in humans hint towards different developmental trajectories of the brain systems underlying boundary-based and intramaze cue-based spatial learning. There is the consensus that hippocampus-dependent spatial learning can already be observed in preschool ages^24–27^ and that navigation performance continues to gradually develop well into school years^28–30^. Although preschool children are already able to use the geometry of 3D spatial boundaries, they still fail to use distances to or relations between landmarks^31,32^ and may still need to recruit substantial extra-hippocampal resources for spatial orientation and navigation. Moreover, younger children have deficits in relying on distal spatial information and therefore show a preference for proximal cues^29,33^. Adult performance levels in building hippocampal-dependent spatial map representations are only reached around the age of 12 years^34^ and the ability to integrate spatial information from allocentric and egocentric reference systems only develops in later childhood or early adolescence^28,35^. In the spatial context, the view-independent allocentric reference system infers spatial locations from spatial layouts i.e., spatial relations between objects, boundaries and other features of the environment, whereas the egocentric reference system infers spatial locations relative to the body axes, and both systems can be tentatively related to the hippocampal- and striatal-dependent spatial learning systems respectively”^30^.

We have previously shown that the relative contributions of hippocampal- and striatal-dependent forms of spatial learning are affected by older age and dopaminergic dysfunction. Relative to younger adults, older adults rely more on a single intramaze cue than on the environment’s boundary during spatial learning^36^. Furthermore, while younger adults’ behavior and brain activity is consistent with the predictions of a computational model that assumes hippocampal-dependent boundary-sensitive spatial processing^37,38^, older adults’ spatial learning is more sensitive to intramaze cue information and more associated with striatal than hippocampal activity^39^. A similar behavioral pattern of prioritizing intramaze cue- over boundary-based spatial learning was observed in older adults with Parkinson’s disease who suffer from a severe degeneration of the dopaminergic pathways^40^. Like healthy older adults, Parkinson’s disease patients are more sensitive to a single intramaze cue when navigating through a virtual environment. However, when under dopaminergic medication, the prioritization of intramaze cue-based over boundary-based learning is reduced^41^, indicating a shift towards more hippocampal-dependent spatial learning when dopamine modulation is  improved. Few studies compared spatial learning during maturation and aging and found aging-related decline in various aspects of spatial learning^42,43^, whereas maturation-related increases in spatial performance seemed to be more pronounced for spatial abilities subserved by the hippocampal formation^42^. To our knowledge, comparisons of spatial navigation performance specifically focusing on hippocampal-dependent boundary processing and striatal-dependent intramaze cue processing across the lifespan are rarely undertaken within one study. The open question is, thus, not whether hippocampal- and striatal-dependent spatial learning are present in childhood and older age, but rather how the relative reliance on using boundary and intramaze cue information may differ between maturation and aging.

To fill these gaps, the current study used a desktop-based virtual navigation task which allows the assessment of relative reliance on the environment’s boundary or a single intramaze cue in a lifespan sample. Based on previous human developmental^28–30^ and aging studies^36,39,44,45^ of spatial learning, we expect a greater prioritization of the intramaze cue over the boundary information in the youngest (6–8 years) and oldest (65–80 years) age groups of our sample. We further assessed working memory performance and intraindividual variability in response times (hereinafter referred to as processing fluctuations) during independent tasks as behavioral correlates of maturation- and aging-related differences in the prioritization of intramaze cue and boundary information during spatial learning. Previous findings showed that prefrontal cortex functions might be involved in the top-down regulation of boundary- and intramaze cue-based spatial learning^5^ and that working memory in particular is implicated in the formation of map-based spatial representations^46,47^. Further evidence indicates a link between working memory and cognitive processing fluctuations: individuals with lower working memory capacity (especially children and older adults) are also more variable in their responses^48^. Processing fluctuations can in turn be related to the efficiency of dopaminergic modulation^49,50^. Individuals with suboptimal dopamine modulation such as children and older adults^17^ also show higher cognitive processing fluctuations^51,52^. Moreover, processing fluctuations seem to be predictive of aging-related cognitive decline in general^53,54^ and hippocampus-dependent episodic memory performance in older age in particular^55^. Finally, working memory capacity might mediate potential effects of a pharmacological dopamine increase during complex tasks requiring top-down model-based control^56^. In light of these previous findings we also investigated the questions to what extent spatial working memory might mediate potential associations between cognitive processing fluctuations and spatial learning and if potential mediation effects may differ during development and aging.

## Methods

### Participants

A total of 155 participants from five age groups, including younger children (YCH, *M* = 7.2 years), older children (OCH, *M* = 11.4 years), adolescents (AD, *M* = 15.9 years), younger adults (YA, *M* = 25.2) and older adults (OA, *M* = 71.0 years), were recruited from a population-based database provided by the city registry of Dresden, Germany. Participants were screened for psychiatric, neurological, and other serious health conditions before study participation. Older adults underwent additional cognitive screening using the Montreal Cognitive Assessment (MOCA)^57^, with a cut-off score of 26 or higher (*M* = 27.9, *SD* = 1.3, range 26–30) as an inclusion criterion. In total, 11 individuals (1 YCH, 1 OCH, 1 AD, 4 YA, 4 OA) were excluded based on these health and cognitive screening criteria. Three additional older adults did not complete the experimental task and were thus excluded from statistical analyses. Thus, the final effective sample of the study included 141 participants, with 27 younger children, 30 older children, 29 adolescents, 29 younger adults, and 26 older adults. Participants received 7.50 Euro per hour for compensation. Ethic approval in accordance with the Helsinki declaration was granted by the ethic committee of the TU Dresden, Germany (EK 354092013). All participants (and parents in the case of children) signed informed consents before the start of study participation.

Demographic characteristics, basic cognitive covariates, and prior experiences with computer games and spatial skills are summarized in Table 1. Gender distribution did not differ between the five age groups. Also, the two adult age groups did not differ in total years of education (*t*(50) = 1.4, *p* = 0.2, *d* = 0.4). Furthermore, all participants reported to have normal or corrected-to-normal vision. Two computerized tasks, the Spot-a-Word (SAW) task and the Identical-Pictures (IDP) task, assessed crystalized and fluid components of basic intellectual abilities, respectively^58^. In line with previous data from a population-based lifespan sample^59^, verbal ability assessed with the SAW task increased linearly from young childhood to older adulthood in this sample (*F*(4,133) = 74.8, *p* < 0.0001, *η*^2^ = 0.7; confirmed linear contrast: *F*(1,133) = 289.5, *p* < 0.0001, *η*^2^ = 0.7). As for the performance assessed with the IDP task reflecting basic cognitive speed, it showed a maturation-related increase from young childhood to young adulthood, which was then followed by an aging-related decline in the older adult sample, *F*(4,133) = 83.7, *p* < 0.0001, *η*^2^ = 0.7; confirmed quadratic contrast: *F*(1,133) = 216.6, *p* < 0.0001, *η*^2^ = 0.6). This inverted U-shaped age gradient of basic cognitive speed across the lifespan is also in line with prior evidence^59^.

**Table 1:** Demographic characteristics, cognitive covariates, and computer/video gaming experience by age group.

	Age group					Test statistic	Effect size
	6–8	10–13	15–17	20–35	65–80		
**Sample characteristics**							
*N*	27	30	29	29	26		
Age (years)	7.2 (0.7)	11.4 (1.0)	15.9 (0.8)	25.2 (4.4)	71.0 (5.5)	*F*(4,136) = 1777.1*	*η*^2^ = 0.98
Gender (F/M)^a^	12/15	14/16	14/15	13/16	13/13	*χ*^2^(4) = 0.2	
Education (years)	1.9 (0.8)	6.1 (1.1)	10.2 (1.1)	16.6 (2.4)	15.5 (3.2)	*F*(4,133) = 300.6*	*η*^2^ = 0.90
**Basic cognitive abilities**							
Spot-a-Word	1.5 (1.8)	9.6 (5.8)	15.3 (5.7)	19.1 (5.4)	23.2 (5.4)	*F*(4,133) = 74.8*	*η*^2^ = 0.69
Identical-Pictures	15.6 (3.8)	23.9 (4.4)	33.5 (4.0)	34.0 (5.5)	23.2 (4.0)	*F*(4,133) = 83.7*	*η*^2^ = 0.72
**Gaming experience**							
none (%)	27	10	25	59	65	*χ*^2^(8) = 41.6*	
< 30 min/day (%)	50	40	14	31	15		
≥ 30 min/day (%)	23	50	61	10	20		

Scores represent means and standard deviations (in parenthesis). Years of education are computed as the sum of school, university, and other education years. The Spot-a-Word (SAW) and the Identical-Pictures (IDP) test scores represent the mean numbers of correct responses (see text for between age group differences in some of these measures); ^a^gender refers to sex without information about gender identity; *p < 0.05.

Furthermore, we assessed the participants’ previous experience with computer/video games (i.e., hours of playing computer/video games per day) in the entire sample as well as profession- and leisure-related experiences with activities that require specific navigation skills (airplane navigation, ship navigation/sailing, other professions/activities) in the two adult age samples. Altogether 37% of the participants reported having no computer/video gaming experiences, 30% reported to play only less than 30 min per day, and 33% played computer/video games more extensively (i.e., at least 30 min per day). The age groups differed in the duration of regular computer/video gaming (*χ*^2^(8) = 41.6, *p* < 0.0001; see Table 1): More extensive gaming experience were reported in the older children and the adolescent group, whereas about 60% of the adult age samples reported not playing computer/video games at all. Of note, there was no difference between the two adult subsamples i.e., between younger and older adults in this regard (*χ*^2^(2) = 2.3, *p* = 0.3). Furthermore, younger and older adults also did not differ in the frequency of pursuing other professional or leisure activities that require specific spatial skills, namely aviation (*χ*^2^(1) = 0.9, *p* = 0.3), ship navigation (*χ*^2^(1) = 0.006, *p* = 0.9) and other navigation activities (*χ*^2^(1) = 1.9, *p* = 0.2).

### Spatial navigation task

We applied a desktop virtual spatial navigation task^5^ that assesses the participants’ reliance on hippocampal boundary-based and striatal intramaze cue-based spatial learning^39,41^. The virtual environment of the task was programed using UnrealEngine2 Runtime software (Epic Games; http://udn.epicgames.com). The virtual environment consisted of a circular grass arena surrounded by a stone wall that was low enough for the participants to have a 360-degree panoramic view of the mountains, clouds, and the sun that served as distal orientation cues in the background, but not as cues for estimating distances and relative locations of objects: the principle of parallax could not be applied to estimate the distance to an object in the virtual arena based on the view of distal background information alone, since all distal cues were plotted at infinity. Within the grass arena a traffic cone was positioned at a fixed location during spatial encoding and learning to serve as intramaze cue. Participants navigated through the virtual arena within the stone wall boundary using a 360-degree joystick. Virtual distances were defined in virtual meters (1 vm = 62.5 UnrealEngine2 units) and the participant’s virtual positions within the virtual arena were sampled continuously with a sampling rate of 10 Hz.

**Figure 1:**
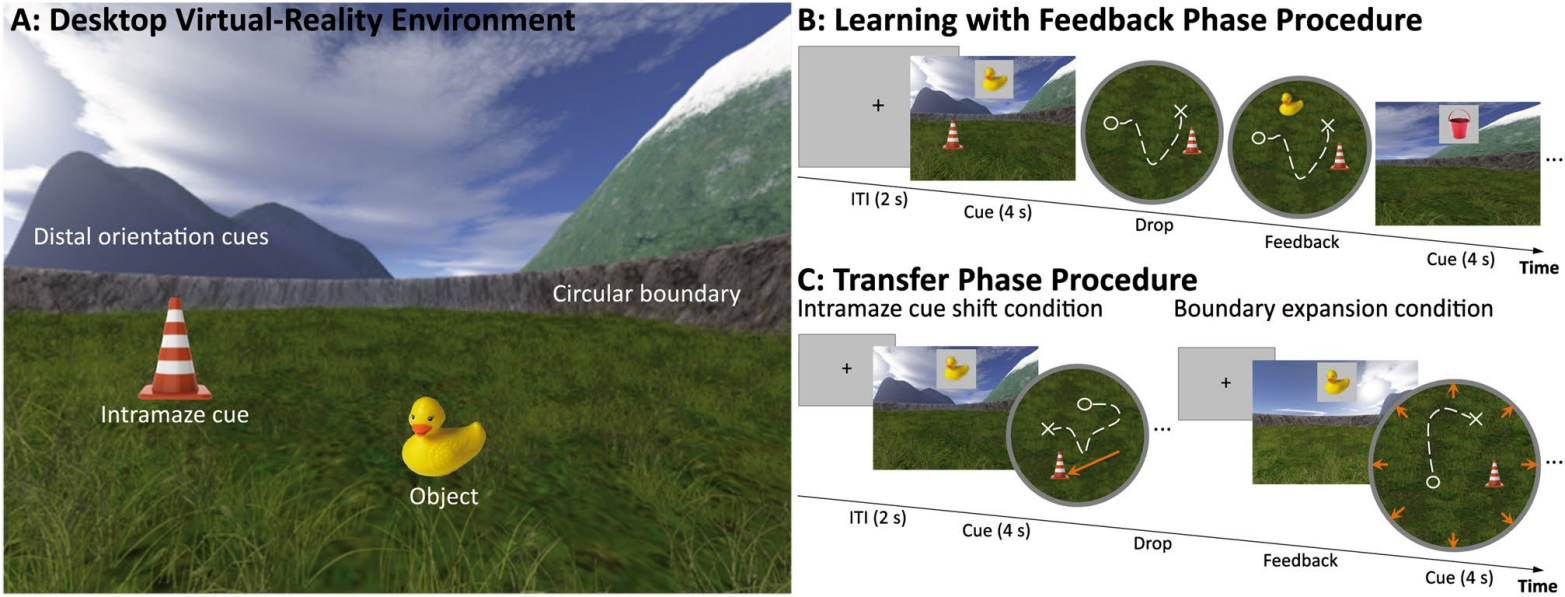
Schematic diagram of the desktop virtual-reality environment (**A**) and experimental procedures of the learning phase with feedback (**B**) and the transfer phase (**C**) with the intramaze cue shift and the boundary expansion condition for manipulating striatal- versus hippocampal-dependent spatial learning, respectively. The white dashed line represents possible movement paths with the white circle indicating a starting point and a white cross indicating a memorized location where the participant placed the respective object by pressing a button (see text for details).

The spatial navigation task comprised three phases in a consecutive order: initial exploration and encoding (phase 1), learning with feedback (phase 2), and transfer (phase 3; see Fig. 1). *Phase 1 (encoding):* During the initial exploration and encoding phase, four everyday objects of different categories were placed one after the other into the virtual arena. We used two different object lists which were counter-balanced between participants and age groups. List one comprised an alarm clock, a basketball, a briefcase, and an accordion and list two comprised a rubber duck, a straw hat, a baguette, and a bucket. Participants were instructed to explore the arena using the joystick (without time limitation), find and memorize each object’s spatial location. Before the next object was presented, participants had to collect the current object by virtually walking over it. *Phase 2 (learning with feedback):* During the following three learning trials, each object was probed once sequentially in a pseudorandom order by presenting a 2D picture of the respective object for four seconds at the center of the upper half of the screen (altogether 12 probes in phase 2). The participants’ task was to navigate back to the memorized location using the joystick and to indicate the respective object’s position (i.e., to virtually drop the object) by pressing the space bar on the computer keyboard. After the participants’ response, a visual feedback was provided i.e., the respective object was shown at its correct location in the grass arena. Participants were asked to collect the object from the feedback position in order to proceed to the next probe. The feedback allowed the participants to correct their memory about the object positions and to improve their performance during phase 2. If participants remembered an object location correctly (i.e., within a 5 vm radius around the correct object coordinates), the feedback “Perfect!” was shown on the screen for three seconds and participants proceeded to the next probe immediately. *Phase 3 (transfer):* The learning with feedback phase was followed by the transfer phase with two independent conditions. In the condition of boundary expansion, the surrounding stone wall boundary was enlarged by 20% of the original radius from 80 to 96 vm, whereas in the condition of the intramaze cue shift the position of the traffic cone was shifted by 30 vm from its original position. Both manipulations were carried out independently i.e., boundary and intramaze cue information was never changed together within one trial and the remaining features of the virtual environment remained the same, including the distal orientation cues. During the transfer phase, each object was probed once in each of the two conditions in a pseudorandom order (altogether 8 probes in phase 3). The participants were again instructed to navigate to the memorized location and to indicate the respective object’s position by a button press without being informed about the changes in the environment. In contrast to the learning phase, no feedback was provided and participants proceeded to the next probe immediately after their response. Throughout all three task phases, the relative size and position of the distal orientation cues were never manipulated and, hence, the information provided by the distal orientation cues remained the same also throughout all trials that either manipulated the boundary (boundary condition) or the intramaze cue information (intramaze cue condition). During phase 1 (encoding), participants started each trial at the center of the virtual arena with a random heading direction, which was not the case in phases 2 (learning with feedback) and phase 3 (transfer). Instead, during phase 2 and 3 participants navigated continuously within each trial. Consequently, the visual information and starting points constantly changed in a trial and participants were able to approach features of the environment (the intramaze cue and the boundary) as well as the memorized object locations from various directions.

Of note, the two conditions of the transfer phase are not to be confused with “transfer conditions” commonly included in cognitive training studies. Instead, the transfer phase in this task is designed to assess the relative sensitivity to boundary or intramaze cue information during spatial navigation and not to assess a potential transfer of learned abilities or strategies to other spatial abilities or task-unrelated cognitive domains. Both types of information differ with regard to behavioral flexibility and error-proneness, which is related to the different underlying brain structures and resources. Whereas hippocampal boundary-sensitive cells respond to changes in geometric parameters of a familiar environment such as the radius of the surrounding boundary^38,60^, learning spatial locations based on a single intramaze cue is rather subserved by the striatum^61,62^. The intramaze cue-based learning is less resourceful but also less flexible or more error-prone compared to boundary-based learning in changing environments or task setups^63,64^. Systematically manipulating the boundary or the intramaze cue as implemented in our task, can provide a measure of the relative prioritization of using hippocampal- or striatal-dependent information during spatial learning and memory, respectively^5,39,41^. We therefore focused on the question how maturation and aging influences the relative prioritization (or brain resources) of intramaze cue-based and boundary-based spatial learning. If a participant relied more on the boundary to infer the objects’ positions during the learning phase, a radial expansion of the boundary during the transfer phase should result in a relative radial shift of the memorized object locations. Therefore, a boundary model which is based on information about the radius expansion should account for the behavioral data better. In contrast, if a participant relied more on the intramaze cue to infer the objects’ locations during the learning phase, shifting the intramaze cue’s position should rather result in a relative shift of the memorized object locations in accordance to the new position of the intramaze cue and a model based on the intramaze cue shift should account for the behavioral data better (for further details on the computational model approach see below).

### Performance measures of spatial navigation

Three types of performance measures were computed from the behavioral data. Performance in the learning with feedback phase was assessed by computing the Euclidian distance (in vm) between the correct and the memorized object locations, with larger distances indicating worse performance. The “correct” object locations were positions denoted by the 2D x-/y-coordinates of the objects as they were actually shown to the participants during the encoding phase (phase 1), whereas the memorized object locations were the x-/y-coordinates of the positions where the participants had placed the objects based on their memory in the learning phase (phase 2) when probed to navigate to the positions of the objects. In contrast, the outcome measures of the transfer phase (phase 3) do not focus on accuracy in terms of the retrieved object locations (x-/y-coordinates) but on the relative sensitivity of the participants to boundary information (boundary condition) or intramaze cue information (intramaze cue condition). To this end, performance in the transfer phase was assessed with model-based estimates. Specifically, to quantify the reliance of navigation performance to either boundary or the intramaze cue, behavioral data from the transfer phase were compared to predictions of two simple geometric models^39^ that utilized either information about the radial expansion of the arena (boundary model applied to the boundary condition trials) or the replacement of the intramaze cue (intramaze cue model applied to the intramaze cue condition trials). The resulting angle measures for the boundary and the intramaze cue condition reflect the similarity between two vectors (a vector connecting the participants’ memorized object positions in the initial environment (from phase 1 and 2) and the changed environment (phase 3) and a vector connecting model-predicted positions of an object in the boundary expansion trials or the intramaze cue shift trials, respectively: the smaller the angle the more similar is the behavioral data to the prediction of either of the two models.

These simplified models were modified after an earlier boundary vector model of hippocampal place cell firings in squared environments^38^ in order to integrate the multitude of directions in circular environments^39^. In a nutshell, the boundary model corresponds to a geometric transformation of each object position **p** to a predicted memorized object position ${\tilde{\mathbf{p}}}_{m}$ after the boundary was enlarged (boundary condition of phase 3), according to the radial change in radius $\Delta r$:

1 $${\tilde{\mathbf{p}}}_{m} = \left( {1 \pm \frac{\Delta r}{{r^{2} }} \left| {\mathbf{p}} \right|} \right){\mathbf{p}}$$

The intramaze cue model assumes that the distance between intramaze cue and object location is kept constant even when the position of the intramaze cue is shifted (translated) by an arbitrary translation vector ($$v).$$ To capture the participants’ performance after the shift of the intramaze cue, the intramaze cue model assumes that the memorized object location ($${\tilde{\mathbf{p}}}_{m} )$$ will be shifted in the same direction as the shifted intramaze cue. Accordingly, the distance between an object position **p** and the intramaze cue can be described by the translation vector: $$\user2{v }{ = }\user2{ p}_{{{\varvec{LC}}}} - {\varvec{p}}$$, such that the distance and direction are given by

2 $${\Delta }_{LC} = \left| v \right| \;{\text{and}}\; \theta_{LC} = tan^{ - 1} \left( {{\raise0.7ex\hbox{${y_{v} }$} \!\mathord{\left/ {\vphantom {{y_{v} } {x_{v} }}}\right.\kern-\nulldelimiterspace} \!\lower0.7ex\hbox{${x_{v} }$}}} \right).$$

The empirical data from the two manipulations in the transfer phase (boundary and the intramaze cue condition) were compared to predictions of the boundary or the intramaze cue model in two steps: First, the model-predicted memorized position for each object after boundary expansion (boundary condition in phase 3) or intramaze cue shift (intramaze cue condition in phase 3) was computed as described above. Second, the directional shifts (in angle degrees) that were predicted by the models to occur after the two independent environmental manipulations were then computed as the angle of the vector that connect the model-predicted memorized position $\left( {{\tilde{\mathbf{p}}}_{m} } \right)$ in phase 3, and the object’s original position (**p**) from phase 1. The empirically observed directional shifts after the environmental manipulations were computed as the angle of the vector connecting the position observed in the behavioral data in the respective condition $({\tilde{\mathbf{p}}}_{o} )$ in phase 3 and the original object locations (**p**) from phase 1. The relative prioritization of (sensitivity to) boundary or intramaze cue information during spatial navigation was then evaluated as the degree of mismatch (i.e., the size of the angle) between the directional shifts in the observed behavioral data and the predictions from the boundary or intramaze cue model (ranging from 0–180°), respectively. A larger mismatch would indicate that the spatial navigation behavior was less sensitive to the boundary or to the intramaze cue.

Lastly, for each participant the difference between mismatches between the empirically observed directional shifts and the model predictions (boundary minus intramaze cue) was computed as an index for individual differences in the relative prioritization of boundary or intramaze cue information during spatial learning. A larger difference indicates that an individual relies more on intramaze cue over boundary information, which is considered to be computationally less demanding (less costly with regard to cognitive resources) during spatial processing.

### Measures of spatial working memory for location and sequence

Besides the spatial navigation task, we also assessed working memory measures of spatial location and serial order by a modified version of a spatial working memory (WM) task developed by Klingberg and colleagues^65,66^. In this task, a 4 × 4 grid of spatial locations marked with white circles was presented on a blue background screen. Following a fixation period of 1000 ms, a sequence of white dots was shown sequentially (600 ms per dot, with a 400-ms inter-stimulus interval) in specific locations in the grid. The number of dots in a sequence were either four or seven, which manipulated the WM load. After a sequence of four or seven dots was presented, one out of the 16 available spatial locations in the grid was probed by highlighting the respective circle. To assess working memory for spatial location, the participants were first instructed to respond by pressing the “yes” or “no” key to indicate whether a dot of the previous sequence was presented at the highlighted location in the grid. If the participants responded “yes”, a digit (1 to 4 or 1 to 7 depending on memory load) was presented at the highlighted location and the second task of the participants was to respond whether or not the digit represented the correct sequential order of the dot in the sequence. The second task assessed spatial serial order memory. If participants responded “no” in the location memory task, the task proceeded immediately to a new sequence of dots. The response deadline was 5000 ms. The task comprised two blocks with a set size of four dots and two blocks with a set size of seven dots. The blocks were presented with a constant 4–7–7–4 order for all participants. Each block included 24 trials, and altogether the task took about 20 min. Response times and accuracy were recorded. A uni-weighted composite score of spatial working memory was computed by averaging z-transformed accuracy scores across both memory types and loads.

### Measure of intraindividual cognitive processing fluctuations

Intraindividual fluctuations in cognitive response times are predictive of task demands and individual differences in cognitive resources^53,59,67^. Furthermore, empirical^49,50,68^ and theoretical evidence^69^ suggests that fluctuations in cognitive response time might serve as a behavioral index of neuronal noise that is modulated by age, the dopamine system and the interaction of both as well as by gray and white matter changes across the lifespan^49^. In line with this evidence and of specific interest to our study, numerous empirical studies reported a maturation- and aging-related increase in cognitive response time fluctuations^51,52^. In our study, trial-by-trial response time fluctuations are referred to as cognitive processing fluctuations and are computed as the standard deviation of response times across trials within each participant during the Identical-Pictures task (IDP)^58^. During this simple visual processing task, simple black-and-white line drawings are presented on the computer screen. The participants were instructed to match the target stimulus that is presented at the center of the upper half of the screen to one of the five stimuli presented below by pressing the respective stimulus number (1 to 5) on the keyboard as fast and as accurately as possible. The task is limited to a duration of 80 s.

### Study procedures

All participants took part in a 1.5- to 2-h session in a laboratory setting. They first completed the sociodemographic questionnaire, followed by the Identical-Pictures and the Spot-a-Word tasks. Before starting the spatial navigation task, participants received a brief joystick training to get familiar with the joystick control in a desktop virtual-reality environment. Only after the participants felt sufficiently confident in using the joystick, the spatial navigation task was started, followed by the spatial working memory task.

### Data analysis

Statistical analysis was carried out using IBM SPSS software (version 25). The demographic sample characteristics and cognitive covariate data were analyzed using univariate analysis of variance (ANOVA) for continuous variables and Pearson Chi-square (*χ*^2^) test for discrete variables. Pairwise age group comparisons were conducted using two-tailed Student’s t-test.

Age group differences in spatial navigation performance were analyzed in separate ANOVA models for the learning with feedback phase (average distance error) and the transfer phase (mismatches between observed behavior and model predictions). Age Group (YCH, OCH, AD, YA, OA) and Gender (females, males) were entered as between-subject factors into the models. The repeated measurement ANOVA model of the transfer phase data further included condition (intramaze cue shift vs. boundary expansion) as the within-subject factor. To check for potential effects of computer/video gaming experiences on age differences in spatial learning and memory, additional analyses were undertaken in two steps: First, we also added the average duration of daily computer/video gaming (none, < 30 min/day, ≥ 30 min/day) as a between-subject factor into the ANOVA models. Second, hierarchical regression analyses were conducted, entering also gender and duration of daily computer/video gaming as predictors. Daily computer/video gaming was entered as dummy coded categorical variable into the regression with ‘no computer/video gaming’ as the reference category and ‘computer/video gaming of less than 30 min per day’ as well as ‘computer/video gaming of 30 or more minutes per day’ as independent variables. The potential effects of age were controlled by partialling out the quadratic age trends across the lifespan from the relevant performance measures using the curve fitting function in SPSS. Thus, standardized T-scores (*M* = 50, *SD* = 10) of the age-partialled residuals of navigation performance from the learning with feedback phase and the transfer phase (computed as difference score between mismatches the boundary and the intramaze cue models) were included as dependent variables into two separate hierarchical regressions.

For the spatial working memory task, age group differences were examined using a repeated measurement ANOVA with Age Group as the between-subject factor and memory Type (location or sequence) as well as memory Load (set size 4 or set size 7) as within-subject factors. Age group differences in the intraindividual variability of cognitive processing time (i.e., cognitive processing fluctuations) during the Identical-Pictures task were examined using univariate ANOVA with Age Group as between-subject factor. In addition, models were reconducted with Gender (females, males) entered as additional between-subject factor.

Additionally, a priori linear and quadratic contrasts were defined for the 5-level between-subject factor Age Group for the ANOVA models of all three tasks using the SPSS function for planned polynomial comparisons. Post-hoc analyses were conducted with second level ANOVA analyses and pairwise comparisons using the Student’s *t*-tests. When applicable, post-hoc pairwise comparisons are reported with Bonferroni corrected p-values or in case of violations of the variance homogeneity assumption as indicated by the Levene’s test, the Games-Howell test statistics were used instead.

Furthermore, correlational and mediational analyses were conducted to investigate the relations between individual differences in the prioritization of intramaze cue- over boundary-based learning, cognitive processing fluctuations and spatial working memory during maturation and aging. For this purpose, a child development subsample (comprising YCH, OCH, AD) and an adult development subsample (comprising YA, OA) were created based on previous literature indicating that although complex allocentric representations of space are available around the age of 12 years^34^, allocentric navigation performance seems to remain below the level of young adult performance throughout adolescence^70^. Both analyses were conducted using the standardized T-scores of the age-partialled residuals of the spatial navigation score reflecting intramaze cue- over boundary-based learning, the standardized T-scores of the age-partialled residuals of the score reflecting cognitive processing in the IDP task, and the standardized T-scores of the age-partialled residuals of the spatial working memory composite score. Analyses were computed separately for the child development (YCH, OCH, AD) and the adult development (YA, OA) subsamples in two steps: First, bivariate Pearson’s product moment correlations were computed for the relative prioritization of intramaze cue- over boundary-based learning with cognitive processing fluctuations and spatial working memory, respectively. Second, for the mediational analyses, the intramaze cue- over boundary-based learning score was entered as dependent variable, whereas cognitive processing fluctuations were entered as independent variable and spatial working memory was entered as mediator variable. Regression-based mediation analysis was conducted using model 4 of the SPSS/SAS toolbox PROCESS version 2.16.3 (afhayes@processmacro.org; see Supplemental Material for further details)^71^. Altogether, 5000 bootstrap samples were applied to estimate the bias-corrected confidence intervals (level of confidence = 95%). In order to gain information on the specificity of the results of the primary mediation model, a second mediational analysis was conducted reversing the assignment of the independent and the mediator variable i.e., in the alternative model spatial working memory was entered as independent variable and processing fluctuations were entered as mediator variable.

Normal distribution of model residuals was examined using the Shapiro–Wilk test and by visual inspection of the Q-Q plots where necessary. Effect sizes for *t*-tests are given as Cohen’s *d*. Effect sizes for ANOVA models are given as partial eta-squared (*η*^2^). The level for statistical significance was set to alpha (*α*) = 0.05 for all statistical tests.

## Results

### Lifespan age differences in spatial memory

**Figure 2:**
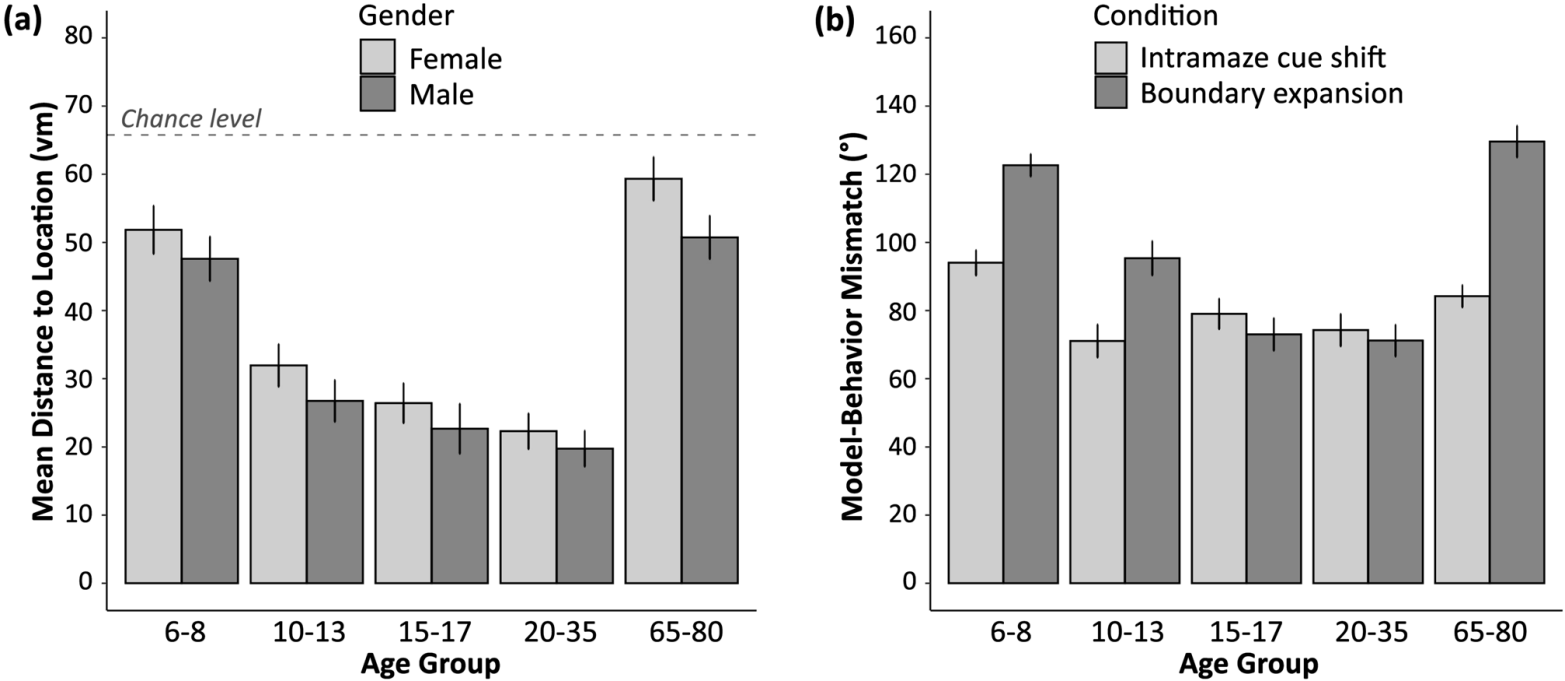
Differential age differences in spatial navigation performance across the lifespan. (**a**) Mean distance between the actual and the memorized object positions during the learning with feedback phase for all five age groups (compared to chance baseline) separated by gender (higher values indicate larger distance errors during spatial learning and memory). (**b**) Angle deviation i.e., mismatch between model predicted and observed behavior in both conditions of the transfer phase (larger angle deviations indicate less intramaze cue- or boundary-based learning during spatial navigation and angles between 90–180° indicate that the model-predicted and the observed vectors point into opposite directions i.e., model-prediction and observed behavior diverge more strongly). Error bars indicate one standard error of the mean.

Data from the learning phase (distance error between the memorized and actual object locations) were examined using ANOVA. Other than Age Group (YCH, OCH, AD, YA, OA), Gender was also included as between-subject factor into the ANOVA model. As shown in Fig. 2A, the analysis revealed significant main effects for Age Group (*F*(4,131) = 47.2, *p* < 0.0001, *η*^2^ = 0.6) and Gender (*F*(1,131) = 6.6, *p* = 0.01, *η*^2^ = 0.05). No interaction was observed, which indicated that spatial memory was better in male participants in all five age groups. Irrespective of gender, spatial memory followed an U-shaped age gradient across the lifespan (*F*(1,136) = 169.4, *p* < 0.0001, *η*^2^ = 0.6), with the youngest children and older adults performing comparable to each other, but worse than all other age groups (*p*s < 0.0001).

To check for potential effects of individual differences in gaming experience on task performance, an additional ANOVA analysis that added the variable of daily duration of computer/video gaming (none, < 30 min/day, ≥ 30 min/day) as an additional between-subject factor was conducted. No main effect of gaming experiences was observed, the main effects of Age Group and Gender were also unaffected. Furthermore, linear regression models were conducted for the child and adult development subsamples. Gaming experience was not predictive of spatial navigation performance in either subsamples (*p*s > 0.1).

### Relative prioritization of intramaze cue- and boundary-based learning

Data from the transfer phase were analyzed using a repeated-measure ANOVA model, with Condition (boundary expansion, intramaze cue shift) as the within-subject factor, Age Group (YCH, OCH, AD, YA, OA) and Gender as between-subject factors. The results revealed significant main effects for Age Group (*F*(4,131) = 23.7, *p* < 0.0001, *η*^2^ = 0.4), Gender (*F*(1,131) = 7.3, *p* = 0.008, *η*^2^ = 0.05), and Condition (*F*(1,131) = 53.1, *p* < 0.0001, *η*^2^ = 0.3) as well as a significant Age Group × Condition interaction (*F*(4,131) = 15.1, *p* < 0.0001, *η*^2^ = 0.3). Although the mismatch between model predicted and observed behavior in both conditions was overall larger in female compared to male participants, no Gender × Condition or Gender × Age Group interaction was found (*p*s > 0.3). Thus, gender was not considered further in the correlational and mediational analyses.

Although intramaze cue- and boundary-based learning (as reflected in the mismatches between observed behaviors and model predictions) followed a quadratic trend across the lifespan (as shown in Fig. 2B), the effect size was much larger when the boundary of the spatial arena was changed (*F*(1,136) = 122.9, *p* < 0.0001, *η*^2^ = 0.5) than when the intramaze cue was shifted (*F*(1,136) = 10.1, *p* = 0.002, *η*^2^ = 0.07). Accordingly, second level analyses of the Age Group × Condition interaction revealed that except for the younger children who showed less intramaze cue-based learning than younger adults (*p* = 0.02), there was no difference regarding this form of learning between the remaining four age groups. In contrast, boundary-based spatial learning yielded an inverted U-shaped function. In particular adolescents and younger adults showed greatly reduced differences between their behavior and the predictions of the boundary vector model, compared to all other age groups (*p*s < 0.02). Younger children and older adults, in contrast, showed a more pronounced deviation from the model predictions (*p*s < 0.0001; *η*^2^ of the quadratic trend = 0.5). Taken together this indicates that the ability to use the intramaze cue for representing simple stimulus–response associations seems to be already well-developed in older children and remains the same thereafter across the lifespan until old age. The ability to represent spatial locations relative to the environmental boundary, in contrast, develops more gradually, reaches adult-level performance only after peri-adolescence, and undergoes a very clear aging-related decline in old age. In line with these findings, adolescents and younger adults show equivalent levels of intramaze cue- and boundary-based spatial learning (*p*s > 0.3). Younger and older children, as well as older adults, however, showed a clear preference for relying on the computationally less costly striatal intramaze cue-based learning, rather than on more demanding hippocampal, boundary-based learning (*p*s < 0.0001).

Main model descriptions and results for all factors and interaction (including non-significant results) for both the learning with feedback phase (phase 2) and the transfer phase (phase 3) are provided in Supplemental Table S1.

We again checked for potential effects of previous computer/video gaming experiences on the above observed results by adding daily duration of gaming (none, < 30 min/day, ≥ 30 min/day) as a between-subject factor into the model. Similar to results for the learning phase, no main effect of gaming experience (*p* = 0.1) or interactions with other factors were observed (*p*s > 0.4). The above reported main effects of Age group, Gender, and Condition as well as the Age Group × Condition interaction all still remained significant after including the additional factor. Based on these results, gaming experience was therefore not considered in further analyses.

### Spatial working memory for location and sequence

Performance accuracy in the spatial working memory (WM) task for each age group is provided in Table 2. Age group differences were examined using repeated measurement ANOVA with WM Type (location, sequence) and WM Load (set size 4, set size 7) as within-subject factors and Age Group (YCH, OCH, AD, YA, OA) as between-subject factor. All groups performed above chance in the different conditions (chance level was 0.5 for location memory and 0.25 or 0.14 for sequence memory in the set size 4 and 7 condition, respectively).

**Table 2:** Cognitive processing fluctuation and spatial working memory performance by memory type (location or sequence), memory load (set size 4 or 7) as well as by age group.

	Age group				
	6–8	10–13	15–17	20–35	65–80
Processing fluctuations^a^	1805.3 (923.7)	983.5 (409.1)	634.1 (199.4)	711.9 (199.6)	928.0 (225.2)
Spatial working memory^b^					
Location (set size 4)	74.4 (14.0)	85.3 (13.7)	97.9 (2.6)	96.2 (3.1)	80.4 (22.9)
Location (set size 7)	63.7 (13.6)	76.2 (13.5)	88.4 (8.2)	88.9 (7.4)	66.9 (16.9)
Sequence (set size 4)	48.9 (20.5)	72.6 (21.7)	89.2 (6.9)	87.3 (12.8)	62.1 (25.4)
Sequence (set size 7)	34.0 (20.2)	57.8 (17.2)	73.9 (8.1)	70.7 (10.5)	44.5 (23.9)

Intraindividual cognitive processing fluctuations are computed as the trial-by-trial standard deviation of the reaction times in the Identical-Pictures task. Spatial working memory scores represent the percentage of mean accuracy with per age group. Standard deviations are provided parenthesis for all mean scores. For age group differences see text. ^a^missing data: n = 2 in the adolescent sample and n = 1 in the older adults sample, ^b^missing data: n = 1 in the young children sample and n = 1 in the older adult sample.

The analysis revealed significant main effects for WM Type (*F*(1,134) = 391.4, *p* < 0.0001, *η*^2^ = 0.7) and WM Load (*F*(1,134) = 213.7, *p* < 0.0001, *η*^2^ = 0.6), indicating, respectively, better mean performance in the location relative to the sequence memory condition as well as better performance with a lower WM load. Furthermore, there was a significant main effect for Age Group (*F*(4,134) = 31.8, *p* < 0.0001, *η*^2^ = 0.5) and a WM Type × Age Group interaction (*F*(4,134) = 9.9, *p* < 0.0001, *η*^2^ = 0.2). Together, these results indicated age differences across the lifespan (confirmed quadratic trends: *F*s(1,134) ≥ 50.7, *p*s < 0.0001, *η*^2^s ≥ 0.3) as well as age differences in the effects of memory type (i.e., the difference between the accuracies in the location minus the sequence memory condition; confirmed quadratic trend: *F*(1,134) = 32.8, *p* < 0.0001, *η*^2^ = 0.2). Performance accuracy was lowest and performance difference between the two memory types was largest in younger children and older adults. Finally, a significant WM Type × WM Load interaction (*F*(1,134) = 19.5, *p* < 0.0001, *η*^2^ = 0.1) indicated that the WM load-related attenuation of performance accuracy was more pronounced for sequence memory than for location memory (*t*(138) =  − 4.5, *p* < 0.0001, *d* =  − 0.4). All other interactions were not significant. The analysis of the spatial working memory task was repeated with Gender as additional between-subject factor. The results revealed no additional main effect of Gender or a Gender × Age Group interaction. Further model details are provided in Supplemental Table S2.

### Intraindividual cognitive processing fluctuations

Age group differences in cognitive processing fluctuations (i.e., intraindividual fluctuations of reaction times measured by the Identical-Pictures task) were examined using univariate ANOVA with Age Group (YCH, OCH, AD, YA, OA) as a between-subject factor. In line with previous findings^59,68^, the results show an U-shaped curve across the lifespan, with a maturation-related reduction of processing fluctuations from younger children to adolescents and an aging-related increase in older adults (*F*(4,133) = 25.9, *p* < 0.0001, *η*^2^ = 0.4). The quadratic function was not symmetric, as fluctuations in older adults were still smaller than fluctuations as in younger childhood (6–8 years; *p*_YCH–OA_ < 0.0001; skewed quadratic contrast: *F*(1,133) = 54.5, *p* < 0.0001, *η*^2^ = 0.3). Intraindividual reaction time fluctuations across trials were comparable in older children and older adults (*p* = 0.97), as well as between adolescents and young adults (*p* = 0.60); whereas younger children showed the highest intraindividual fluctuations of cognitive processing compared to all other age groups (*p*s ≤ 0.001). Again, repeating this analysis with Gender as between-subject factor, revealed no main effect of Gender or Gender × Age Group interaction. For further model details see Supplemental Table  S2.

### Correlational and mediational analyses

To further investigate potential behavioral correlates of differences in the relative prioritization of intramaze cue- over boundary-based spatial learning, correlational and mediational analyses were conducted separately for the child development (YCH, OCH, AD) and adult development (YA, OA) subsamples, using age-partialled measures to control for potential spurious relations. Given that gender did not interact with the primary outcome measure (i.e., the relative prioritization of intramaze cue over boundary information), spatial working memory or cognitive processing fluctuations, we did not consider gender in the mediation analysis.

**Figure 3:**
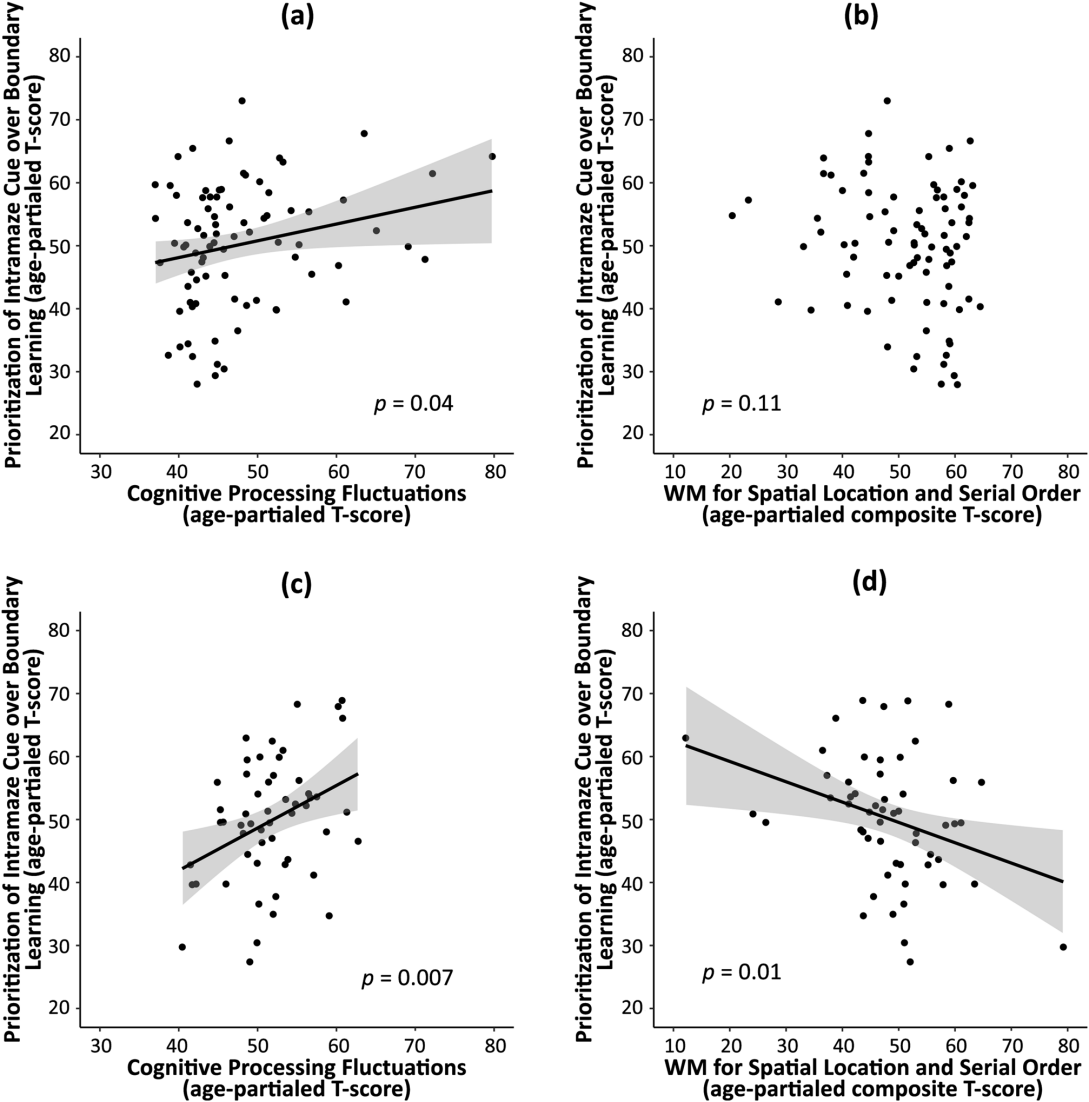
Scatterplots of the bivariate Pearson’s product moment correlations for the child development subsample (panels **a**, **b**) and adult development subsample (panels **c**, **d**). Shown are correlation between cognitive processing fluctuations (panels **a**, **c**) or spatial working memory capacity (panels b,d) and the relative prioritization of intramaze cue-based over boundary-based learning. The gray areas around the linear regression lines represent the 95% confidence intervals of the linear model predictions.

As shown in Fig. 3, linear correlation analyses in the child development subsample indicated that the relative prioritization of intramaze cue-based over boundary-based spatial learning was associated with cognitive processing fluctuation (*r* = 0.23, *p* = 0.04), such that higher processing fluctuations were associated with a greater reliance on the intramaze cue (compare Fig. 3a with 3b). In the adult development subsample, the relative prioritization of intramaze cue-based over boundary-based learning associated both with individual differences in processing fluctuations (*r* = 0.36, *p* = 0.007) and spatial working memory capacity (*r* =  − 0.33, *p* = 0.01). Adults who showed higher processing noise or lower working memory capacity also showed a greater reliance on intramaze cue-based over boundary-based learning during spatial navigation (Fig. 3c, d). The data were carefully checked for outliers and none of the included data points qualified as statistical outliers. Additionally, all correlations were bootstrapped (n = 5000 samples, bias-corrected 95% CI) and none of the correlation results proved to be inconclusive.

**Figure 4:**
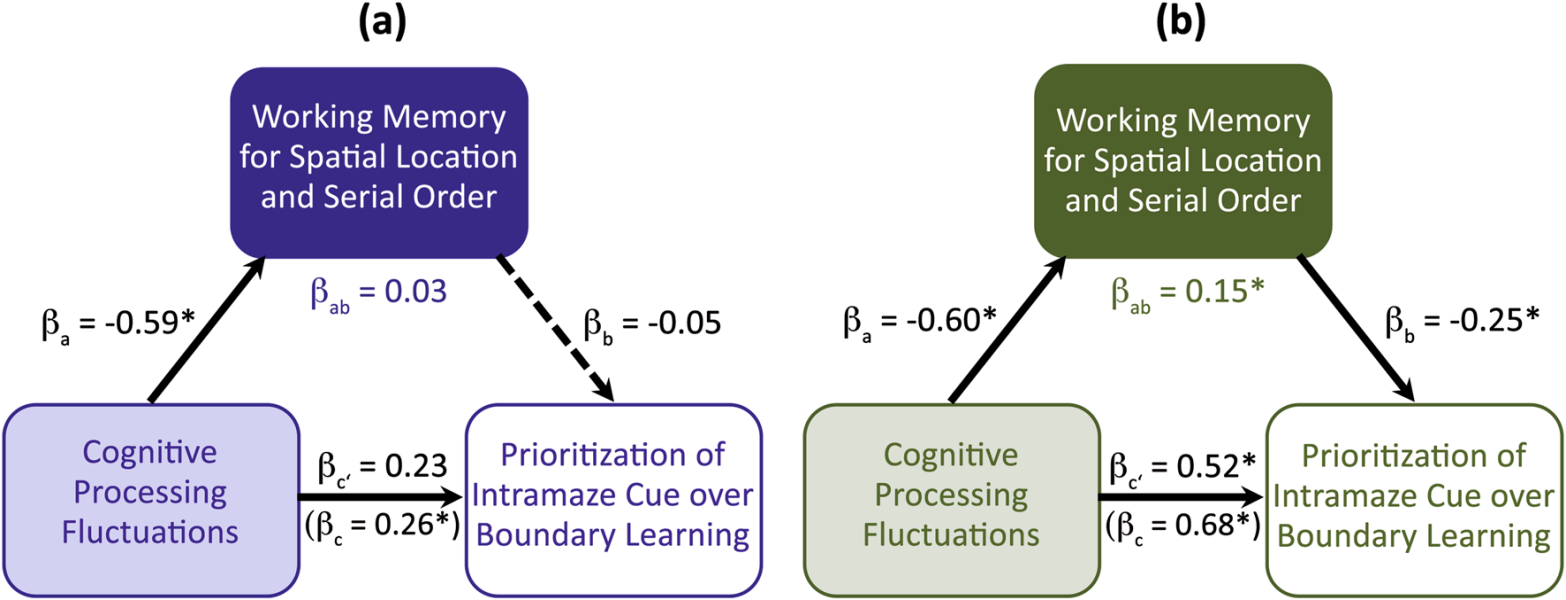
Schematic diagram of the mediational analyses for the child developmental subsample (panel **a**) and the adult developmental subsample (panel **b**). The model tested whether the effect of individual differences in cognitive processing fluctuations (independent variable) in predicting individual differences in the relative prioritization of intramaze cue-based over boundary-based learning is mediated by spatial working memory (mediation variable). Path coefficients are unstandardized beta (β) scores. The β_c′_ scores reflect the direct effect of the independent on the dependent variable when controlling for the mediator variable. The β_c_ scores in parenthesis reflect the total effect (i.e., the sum of the direct and the indirect effect). The partial mediation (β_ab_) only reached significance in the adult development sample (penal b). *p < 0.05.

To further explore these relations in a mediation analysis, we considered cognitive processing fluctuations as the independent variable and spatial working memory as a mediator variable for predicting interindividual variance in the prioritization of spatial learning modes (see Fig. 4 for a schematic overview of the results). Similar to the correlational analyses, the mediational analysis indicates a positive association between cognitive processing fluctuations and the relative prioritization of intramaze cue- over boundary-based learning during child development and aging. In addition, mediational analysis showed in both subsamples that cognitive processing fluctuations predict spatial working memory capacity (*a* path), with high processing fluctuations being associated with low spatial working memory capacity during child development (*β*_*a*_ =  − 0.42, *p* = 0.0001) and adult development (*β*_*a*_ =  − 0.43, *p* = 0.04). In the child development sample (see Fig. 4a) the mediational analyses further showed that the direct effect of cognitive processing fluctuation on the relative prioritization of intramaze cue- over boundary-based learning (*c’* path) narrowly missed significance (*β*_*c′*_ = 0.23, *p* = 0.077) when controlling for spatial working memory capacity despite a significant total effect (*c* path, *β*_*c*_ = 0.26, *p* = 0.02), which might hint towards other mediators that were not investigated in this study. In contrast, in the adult development sample (see Fig. 4b) a complementary partial mediation i.e., a significant *a* × *b* path (*β*_*ab*_ = 0.15, bootstrapped 95% *CI*_lower_ = 0.01 and *CI*_upper_ = 0.46; *β*_*c*_ = 0.68, *p* = 0.01) and a reduced but still significant *c′* path (*β*_*c’*_ = 0.52, *p* = 0.04) could be shown. Accordingly, only in the adult development sample is the relation between cognitive processing fluctuations and the prioritization of intramaze cue over boundary information partially mediated by the individuals’ spatial working memory capacity. In order to gain information about the specificity of the results obtained from the primary mediation model of interest, the assignment of the independent and the mediator variable was reversed in a second model. Mediation in the second model did not reach significance, supporting the preference of the initial model.

Given that processing fluctuations are usually related to mean reaction time and given that mean reaction time in the Identical-Pictures task was also positively correlated with the relative prioritization of intramaze cue- over boundary-based learning during spatial navigation in the adult development subsample (r = 0.35, *p* = 0.009), we repeated the mediation analysis for the adult development subsample with mean reaction time instead of cognitive processing fluctuations as independent variable. The results showed that faster mean reaction times predict better spatial working memory (*β*_*a*_ =  − 0.66, *p* = 0.003). The results further showed that the influence of mean reaction time on the intramaze location cue over boundary prioritization was fully mediated by spatial working memory capacity (*β*_*ab*_ = 0.16, 95% *CI*_lower_ = 0.006, *CI*_upper_ = 0.419; *β*_*c′*_ = 0.37, *p* = 0.1), indicating that the influence of mean reaction time on the intramaze location cue over boundary prioritization seems to be exerted only via the effect of working memory.

## Discussion

Evidence from animal^72^ and human studies^24–26^ indicates that striatum-associated learning of stimulus–response associations and hippocampal learning of spatial layouts can already be observed early in development. Yet, how the prioritization of these two systems during spatial navigation differs across the lifespan is largely unknown. Furthermore, functional correlations of such prioritization with task-unrelated cognitive processes that might contribute to spatial learning and memory performance are not well understood. The current study used a desktop virtual spatial navigation task in combination with model-based behavioral data analyses to investigate the differential prioritization of the two forms of spatial learning across the lifespan in five age groups, covering the age range from 6 to 80 years. Rather than using an age-adjusted task^43^, we used the same task setup in all age groups to better compare navigational performance directly across the lifespan. This approach revealed remarkably different age gradients of intramaze cue-based and boundary-based spatial learning across the lifespan, which in previous work have shown to be subserved by the striatum and hippocampus, respectively^5,39^. Specifically, intramaze cue-based learning reached adult level very early during development in later childhood (between 8 to 10 years) and remained thereafter even into old age. Boundary-based learning, in contrast, showed a more protracted development maturing only after peri-adolescence and exhibiting clear aging-related decline. These findings are in line with previous evidence showing an earlier development of spatial learning guided by intramaze cues rather than allocentric position^73,74^ and a more delayed development of reliable hippocampal place cell maps in animals^21^. The results are further consistent with studies indicating gradual maturation of the ability to learn spatial relations throughout school years in humans^28–30^. The more preserved intramaze cue-based learning in older adults relative to boundary-based learning is also in accord with findings from previous research on the effect of aging on spatial navigation abilities^36,39,44,45^.

Going beyond revealing divergent age gradients of the two forms of spatial learning across the lifespan, the current study provides new evidence for maturation- and aging-related prioritization of intramaze cue over boundary information and sheds new insights on functional correlates of such prioritizations that share similarities but also yield differences between the two life periods of child and adult development. Whereas younger children (6–8 years), older children (10–13 years) and older adults (65–80 years) showed a preference in relying more on the computationally less demanding form of learning, the prioritization of either form of spatial learning does not differ in adolescents (15–17 years) or in younger adults (20–35 years). This might suggest that in matured neurocognitive systems of spatial navigation the relative computational demands of processes play a less important role and that both forms of spatial learning could in principle operate in parallel^5^. These findings might also tentatively hint towards the role of the mPFC in shaping this trajectory, which has been shown to be implicated in regulating striatal- versus hippocampal-dependent strategies in rats^75^ and humans^76^ and which matures throughout late adolescence and young adulthood^77^. Furthermore, the finding that the computationally more demanding boundary-based spatial representation is well developed already in adolescence nicely parallels the result of a study on developmental gradients of habitual and goal-directed reinforcement learning, which showed that the computationally more demanding model-based/goal-directed decision strategy becomes evident in adolescence, whereas the computationally cheaper model-free/habitual decision strategy was apparent in children, adolescents and adults^78^. The pronounced decline of boundary-based learning in older age, on the other hand, is consistent with a similar shift towards computationally less demanding processes observed in decision making^79^ and learning^80^.

In terms of behavioral functional correlates contributing to maturation- and aging-related prioritization of the less demanding intramaze cue-based learning, results from the correlational and mediational analyses point to functions supported by the prefrontal cortex and dopamine modulation. Higher levels of cognitive processing fluctuations, which presumably reflect individual differences in dopamine receptor mechanisms, predicted lower spatial working memory capacity and, importantly, a greater reliance on the less demanding intramaze cue-based learning. The direct link between higher processing fluctuations and a greater reliance on intramaze cue information was found to be stronger in the adult development subsample, whereas during child development the direct link missed significance if spatial working memory was considered as a mediating factor. The rather high variance of intraindividual processing fluctuations especially in the younger children might have contributed to underestimating the direct and indirect association with the relative prioritization of the intramaze cue-based learning in the navigation task in the developmental subsample. In contrast, in the adult subsample, other than the direct link predicting the prioritization of intramaze cue- over boundary-based learning in individuals with higher processing fluctuations, the prefrontal cortex-dependent spatial working memory capacity also partially mediated this effect. These results are interesting as processing fluctuations have been shown to exhibit pronounced changes across the lifespan^59^ and to be associated with dopamine receptor binding efficacy^50^ as well as with brain electrophysiological correlates of frontal control^68^ and thus might represent one underlying factor of the here observed changes. However, based on the results of our additional mediation analysis considering mean reaction time instead of processing fluctuations as independent variable, we assume that the influence of processing fluctuations on the prioritization of intramaze cue- over boundary-based learning might be shared with the effect of perceptual processing speed during adult development. This would also be in line with the finding that dopamine D2 receptor binding in the anterior cingulate cortex and the hippocampus (which are related to working memory and spatial learning, respectively) is not exclusively associated with cognitive processing fluctuations but also with speed^81^.

Evidence from animal research indicates that dopamine modulation of the hippocampus plays crucial roles in regulating the stability (i.e., consistent place-cell firings) of cognitive maps^82^. It is also known that place-specific firings of hippocampal neurons are less consistent (noisier) in older rodents^83^, and besides modulating frontal-striatal executive control processes, dopamine modulation via the hippocampal-striatal loop also regulates habitual and goal-directed spatial exploration^84,85^. Moreover, single unit recordings in rodents showed significant striatal contributions to spatial working memory^86^ and positron emission tomography studies in humans indicate a similar link between striatal (caudate) dopamine and working memory performance^87,88^ as well as a partial mediation effect of striatal dopamine on the effect of adult age on individual differences in working memory tasks^88^. Taking the striatal role in spatial working memory and in the relative prioritization of intramaze cue over boundary information during spatial learning into account might lend further tentative support for the role of dopamine modulation in spatial navigation. Furthermore, the protracted relevance of spatial working memory capacity in mediating the influence of processing fluctuations on the relative prioritization of intramaze cue- vs. boundary-sensitive spatial learning might be related to the protracted maturation of functional networks implicated in spatial working memory^89^. Specifically, the linear increase of spatial working memory capacity from young childhood to late adolescence is associated with increasing white matter maturation and higher working memory task-related activity in frontal and parietal brain regions^90^. In contrast, it has been shown that during adult development spatial working memory positively correlates with spatial learning performance^91^, with the role of spatial working memory in contributing to individual differences in spatial learning depending on the individuals’ abilities to detect and integrate the spatial information provided in the environment^92^. Finally, we must take into account that the results from the correlational and mediation analyses regarding the relation between cognitive processing fluctuations and the prioritization of intramaze cue over boundary information, which was partially mediated by spatial working memory capacity in the adult development sample, was solely derived from young and older adults’ behavioral data. A replication of this mediation effect in a continuous adult development sample, also including middle aged adults, would be of specific interest for future studies investigation spatial navigation in the context of lifespan development.

Taken together, our findings suggest that the differential prioritization of intramaze cue- and boundary-based spatial learning across the lifespan is associated with individual differences in the behavioral functions supported by dopamine modulation of the frontal-hippocampal-striatal circuitry. During child development individual differences in the relative reliance on the two forms of spatial leaning seem less dependent on dopamine modulation of this circuitry, consistent with the idea that anatomical^93^ and functional^94^ connectivity of the hippocampus mature around peri-adolescence. However, during aging the dopamine modulation of this circuitry substantially declines^19^ alongside with clear anatomical^95^ and functional^96^ impairments in these regions. Thus in later life individual differences in the utilizations of different forms of spatial learning seem to become more dependent on dopamine modulation and prefrontal working memory functions.

## Supplementary Information

Supplementary Information.

## Data Availability

The raw data supporting the conclusions of this manuscript will be made available by the authors to any qualified researcher.
