# Supplementary material for: Differential prioritization of intramaze cue and boundary information during spatial navigation across the human lifespan
Source: Sci Rep. 2021 Jul 27;11:15257. doi: 10.1038/s41598-021-94530-9 (PMC8316315; doi:10.1038/s41598-021-94530-9)
Supplement: Supplementary file 1 — Supplementary Information. [file 41598_2021_94530_MOESM1_ESM.pdf]

SUPPLEMENTAL MATERIAL

Differential Prioritization of Intramaze Cue and Boundary Information during Spatial Navigation across the Human Lifespan

Franka Glöckner[1*], Nicolas W. Schuck[2,3], Shu-Chen Li[1,4*]

[1] Faculty of Psychology, Chair of Lifespan Developmental Neuroscience, Technische Universität Dresden, D-01069 Dresden, Germany

[2] Max Planck Research Group NeuroCode, Max Planck Institute for Human Development, D-14195 Berlin, Germany

[3] Max Planck UCL Centre for Computational Psychiatry and Ageing Research, Berlin, Germany

[4] CeTI – Centre for Tactile Internet with Human-in-the-Loop, Technische Universität Dresden, D-01069 Dresden, Germany

* Corresponding authors:
Please address correspondences to:
Franka Glöckner (Franka.Gloeckner@tu-dresden.de), and
Shu-Chen Li (Shu-Chen.Li@tu-dresden.de)
Chair of Lifespan Developmental Neuroscience
Faculty of Psychology, TU Dresden
Zellescher Weg 17, D-01069 Dresden, Germany

ORCID
Franka Glöckner https://orcid.org/0000-0001-9684-7705
Shu-Chen Li https://orcid.org/0000-0001-8409-5390

Methods

Spatial navigation task

Performance measures of spatial navigation (chance performance level). For the learning with feedback phase (phase 2), the chance performance level ($dist_{\text{chance}}$) was computed as the sum of means over the square root of the sum of squares (Euclidian norm) for each object position relative to 10,000 sampled positions within the virtual arena, averaged over the four object locations (see Equation S1). The computation of the chance performance level considered the following two aspects of the task design: (1) the positions of the objects within the circular arena were chosen as such that distances to the arena's center, to the intramaze location cue and to the boundary differed between objects and (2) each object position was chosen as such that distances were *not* equidistant to the arenas center, the intramaze location cue and the surrounding boundary. Given that two different object list were counter-balanced between subjects, chance levels were further averaged for Figure 2a.

Equation S1:

$$dist_{chance} = \frac{\sum_{i=1}^{k} \frac{\mu\left(\sqrt{(X - x_i)^2 + (Y - y_i)^2}\right)}{C}}{k}$$

X, Y – x-and y-coordinates of 10,000 sampled positions in the virtual arena

x_i, y_i – x-and y-coordinates of the four object locations

C = 62.5 (Unreal Units; equivalent to 1 virtual meter)

k = 4 (number of object locations)

Virtual meter units in the spatial navigation task. Virtual distance was measured in Unreal Units (metric scale), which was predefined in the task software Unreal Engine (version UE2). In the reported task version, 1 virtual meter (vm) was equivalent to 62.5 Unreal Units (1 Unreal Unit = 0.625 virtual centimeter).

Data analysis

Mediation analysis using PROCESS model 4. Model 4 assumes a simple mediation model, which we conducted with one independent variable (*X*), one dependent variable (*Y*) and one mediator (*M*). In this model, path *a* refers to the causal association between *X* and *M* (*X* → *M*), path *b* refers to the causal association between *M* and *Y* (*M* → *Y*), and $a \times b$ refers to the indirect effect of *X* on *Y* mediated by *M*. Path *c′* further refers to the direct effect of *X* on *Y* when controlling for *M*, whereas the *c* path reflects the total effect of *X* on *Y* (*X* → *Y*), which is defined as the sum of the direct and the

indirect effects ($c = a \times b + c'$). A complete mediation can be assumed when $a \times b$ is significant and c' is statistically not different from zero (and closer to zero when controlling for *M*). When both $a \times b$ and c' are statistically significant, it can be assumed that *M* only partially mediates the effect of X on *Y*. A mediation can be considered as complementary (or consistent) when $a \times b$ and c' have the same sign (positive or negative). Otherwise a mediation would be considered as competitive (or inconsistent), hinting towards potential suppressor effects.

Results

TABLE S1: ANOVA models for the spatial navigation task phase 2 (learning with feedback) and phase 3 (transfer i.e., intramaze location cue shift vs. boundary expansion)

Model	Factors and interactions	*F*(df1,df2)	*P*	η^2
Navi-Phase-**2**_1	Age Group	*F*(4,131) = 47.2	**< 0.0001**	0.6
	Gender	*F*(1,131) = 6.6	**0.01**	0.05
	Age Group × Gender	*F*(4,131) = 0.3	0.9	0.008
Navi-Phase-**2**_2 (Gaming)	Age Group	*F*(4,110) = 28.0	**< 0.0001**	0.5
	Gender	*F*(1,110) = 6.5	**0.01**	0.06
	Gaming	*F*(2,110) = 0.1	0.9	0.002
	Age Group × Gender	*F*(4,110) = 0.3	0.9	0.01
	Age Group × Gaming	*F*(8,110) = 0.9	0.6	0.06
	Gender × Gaming	*F*(2,110) = 0.4	0.7	0.007
	Age Group × Gender × Gaming	*F*(7,110) = 0.3	0.9	0.02
Navi-Phase-**2**_3 (Quadratic trend)	Age Group: Linear	*F*(1,136) = 0.2	0.7	0.001
	Age Group: Quadratic	*F*(1,136) = 169.4	**< 0.0001**	0.6
Navi-Phase-**3**_1	Age Group	*F*(4,131) = 23.7	**< 0.0001**	0.4
	Gender	*F*(1,131) = 7.3	**0.008**	0.05
	Condition (within-subject)	*F*(1,131) = 53.1	**< 0.0001**	0.3
	Age Group × Gender	*F*(4,131) = 0.4	0.8	0.01
	Age Group × Condition	*F*(4,131) = 15.1	**< 0.0001**	0.3
	Gender × Condition	*F*(1,131) = 1.1	0.3	0.008
	Age Group × Gender × Condition	*F*(4,131) = 0.6	0.7	0.02
Navi-Phase-**3**_2 (Gaming)	Age Group	*F*(4,110) = 13.3	**< 0.0001**	0.3
	Gender	*F*(1,110) = 5.5	**0.02**	0.05
	Gaming	*F*(2,110) = 2.1	0.1	0.04
	Condition (within-subject)	*F*(1,110) = 28.3	**< 0.0001**	0.2
	Age Group × Gender	*F*(4,110) = 0.7	0.6	0.03
	Age Group × Condition	*F*(4,110) = 7.9	**< 0.0001**	0.2
	Age Group × Gaming	*F*(8,110) = 0.4	0.9	0.03
	Gender × Condition	*F*(1,110) = 0.1	0.7	0.001
	Gender × Gaming	*F*(1,110) = 2.9	0.06	0.05
	Condition × Gaming	*F*(2,110) = 0.2	0.9	0.003
	Age Group × Gender × Condition	*F*(4,110) = 0.5	0.7	0.02
	Age Group × Gender × Gaming	*F*(7,110) = 0.6	0.7	0.04
	Gender × Condition × Gaming	*F*(2,110) = 0.5	0.6	0.009
	Age Group × Gender × Condition × Gaming	*F*(7,110) = 1.3	0.3	0.08
Navi-Phase-**3**_3 (Quadratic trend)	Age Group: Linear–Intramaze cue shift	*F*(1,136) = 1.1	0.3	0.008
	Age Group: Linear–Boundary expansion	*F*(1,136) = 0.6	0.5	0.004
	Age Group: Quadratic–Intramaze cue shift	*F*(1,136) = 10.1	**0.002**	0.07
	Age Group: Quadratic–Boundary expansion	*F*(1,136) = 122.9	**< 0.0001**	0.5

Table S1. Note that not all subjects provided information in the gaming experience questionnaire.

TABLE S2: ANOVA models for the working memory (WM) task (spatial location and sequence memory) and for cognitive processing noise (Identical Pictures task)

**Model**	**Factors and interactions**	***F*(df1,df2)**	***P***	η^2
Working-Memory _1	Age Group	$F(4,134) = 31.8$	**< 0.0001**	0.5
	WM Type (within-subject)	$F(1,134) = 391.4$	**< 0.0001**	0.7
	WM Load (within-subject)	$F(1,134) = 213.7$	**< 0.0001**	0.6
	Age Group × WM Type	$F(4,134) = 9.9$	**< 0.0001**	0.2
	Age Group × WM Load	$F(4,134) = 0.6$	0.7	0.02
	WM Type × WM Load	$F(1,134) = 19.5$	**< 0.0001**	0.1
	Age Group × WM Type × WM Load	$F(4,134) = 0.5$	0.7	0.02
Working-Memory_2 (Quadratic trend)	Age Group: Linear–Location/Set Size 4	$F(1,134) = 8.3$	**0.005**	0.06
	Age Group: Linear–Location/Set Size 7	$F(1,134) = 6.7$	**0.01**	0.05
	Age Group: Linear–Sequence/Set Size 4	$F(1,134) = 13.3$	**< 0.0001**	0.09
	Age Group: Linear–Sequence/Set Size 7	$F(1,134) = 11.1$	**0.001**	0.08
	Age Group: Quadratic–Location/Set Size 4	$F(1,134) = 50.7$	**< 0.0001**	0.3
	Age Group: Quadratic–Location/Set Size 7	$F(1,134) = 83.5$	**< 0.0001**	0.4
	Age Group: Quadratic–Sequence/Set Size 4	$F(1,134) = 76.4$	**< 0.0001**	0.4
	Age Group: Quadratic–Sequence/Set Size 7	$F(1,134) = 97.9$	**< 0.0001**	0.4
WM_Difference_3 (Quadratic trend)	Age Group: Linear–Location-Sequence-Diff.	$F(1,134) = 6.4$	**0.01**	0.05
	Age Group: Quadratic–Location-Sequence-Diff.	$F(1,134) = 32.8$	**< 0.0001**	0.2
Processing-Noise_1	Age Group	$F(4,133) = 25.9$	**< 0.0001**	0.4
Processing-Noise_2 (Quadratic trend)	Age Group: Linear	$F(1,133) = 47.4$	**< 0.0001**	0.26
	Age Group: Quadratic	$F(1,133) = 52.3$	**< 0.0001**	0.28
	Age Group: Skewed Quadratic	$F(1,133) = 54.5$	**< 0.0001**	0.29

Table S2. Note that linear trends for the working memory task for spatial location and sequence (model WM_2) as well as the linear trend for age differences in the effects of WM type (i.e., the difference between the accuracies in the location minus the sequence memory condition; model WM_3) also reached significance but with much lower effect sizes. Linear trends are therefore not reported in the main text. For model Processing-Noise_2 we reported the contrast which best described the data and also resulted in the numerically highest effect size (skewed quadratic).
